# Sciential Medicine—A New System of Philosophy

**Published:** 1835-04-01

**Authors:** 


### SCIENTIAL MEDICINE—A NEW SYSTEM OF PHILOSOPHY, &c. By *Thomas Eden*, M.R.C.S.

SOME twenty-five years ago there was published, in eight or ten cantos, a poem called the "CRUISE," by a Lieutenant in the Royal Navy. So exquisitely ridiculous was this poem in its versification, nautical phrases, metaphors, and whole machinery, that it convulsed with laughter the crews (from the cabin to the cockpit) of every ship bearing the British flag! So universal was this effect, that many people began to doubt whether this said naval EPIC was not the production of some extremely clever fellow, who *purposely* rendered it a jest by an affectation of sincerity. It turned out, however, that the author *was in earnest*, and therefore he lost his immortality. Such, we suspect, will be the fate of the author of SCIENTIAL MEDICINE, excepting that part of it which relates to momentary *notoriety*. In this respect, Mr. Eden will be woefully disappointed. There can be little doubt that his expectations are of the most sanguine kind—that he supposed his new philosophy would astound the profession, and produce a kind of *emeute* in the medical world! No such thing will take place. His lucubrations are more insane than the ravings of Rabelais—untinctured with humour, wit, or kind-heartedness. From beginning to end, the book is full of self-conceit and professional malice. The author labours to ridicule and debase the practitioners of medicine, as if no one in that philanthropic department of human knowledge had ever possessed a gleam of light or reason, till Thomas Eden, Esq. Member of the Royal College of Surgeons, came into the world, in order to disseminate his precepts and propositions. And what is this "New System of Philosophy?" A tissue of absurdities—for the most part unintelligible—except where it is libellous of every thing respectable in medicine! A single quotation will justify this severity of censure—a severity in which we rarely indulge, but which will be *felt* when we exercise it.

"284. Honey is best tasted when concrete, folly best seen when concentrate, and individual inabilities are best estimated by observing that many of them amount to nothing. And it is by observing the proceedings of medical societies —it is by observing that the inabilities of professors, when accumulated, amount to impotency, that the *acephalous* nature of the science they study is best appreciated. When Bacon took away the folly of *reasoning without fact*, he left us under the equally great delusion of *fact without reasoning;* and the professors of *acephalous* medicine unable to reason, without the power of inferring, and not knowing that only that knowledge which applies to the future is useful, meet in medical societies, and in solemn conclave talk solely of things which are past, found drivelling questions on by-gone facts,—egotistically communicate their seemings,—all busily intent on mutually vomiting and swallowing one another's opinions. And so utterly impotent is the accumulated intellect of the educated in *acephalous* medicine, that professors of the science meet, and neither by premeditation nor by any accidental oozing does any useful knowledge, session after session, escape from any one of them. So *utterly impotent are their reasoning powers*, that even the verbal sign of mental inference—the word 'therefore'—never occurs in their proceedings; that or any other sign of inference is the 'so be it,' the 'amen,' which ever sticks ominously in the throat of medical professors, —they cannot utter it; or, in the proceedings of medical societies, there are about as many sessions as there are inferences, that is, counting both the right (?) and the wrong, there appears from their accumulated strength about one inference a year. Yet the members of such societies are well satisfied, because they are thoroughly well deluded; they busy themselves with *fact*, and then suppose they have obtained useful *knowledge;* they look into the past, and are confident they are at the truth; but, 'like monkeys at a looking-glass,' they never catch hold of it, and near as it seems, they never can.* And there are now in the metropolis of Britain medical societies who when you ask them their age point to their archives and *talk of centuries*, and yet from the hour of their institution to the present moment, all the useful knowledge they have obtained may find record ample enough on the *head of a shilling*, and permanent enough on the tail of a tadpole. Such is *acephalous medicine*, and such are its professors; things fitly made, and by many of the scientific fitly used for laughing-stocks; and persons who are becoming, and I will bear my testimony 'justly becoming,' a proverb to the people—persons who liberally labour for the public good, and for their blindness amply reap derision." 259.

We shall not waste any more time or space on such an insane and ill-natured production. The man who calmly points out the errors of the profession to his professional brethren, is a benefactor to them and to the community; but he who launches his tomahawk against the *whole profession* is an assassin of character, who deserves no pity and can expect no countenance.

---

* "I advise the medical societies of the metropolis (the medico-chirurgical especially) to publish *all* their facts; and as no useful knowledge—no useful 'living' truths—ever came out of their past inert facts, as a suitable title to them, or motto, I recommend these two words—'*Addled Eggs*.'"

